# Comparative Reinforcement Effect of *Achatina fulica* Snail Shell Nanoparticles, Montmorillonite, and Kaolinite Nanoclay on the Mechanical and Physical Properties of Greenpoxy Biocomposite

**DOI:** 10.3390/polym14030365

**Published:** 2022-01-18

**Authors:** Oluwatoyin Joseph Gbadeyan, Sarp Adali, Glen Bright, Bruce Sithole

**Affiliations:** 1 School of Engineering, Discipline of Mechanical Engineering, University of Kwazulu-Natal, Durban 4041, South Africa; adali@ukzn.ac.za (S.A.); brightg@ukzn.ac.za (G.B.); 2 School of Engineering, Discipline of Chemical Engineering, University of Kwazulu-Natal, Durban 4041, South Africa; Bsithole@csir.co.za; 3 Biorefinery Industry Development Facility, Council for Scientific and Industrial Research, Pretoria 0001, South Africa

**Keywords:** *Achatina fulica* snail shell nanoparticle, montmorillonite, kaolinite, mechanical properties, water absorption

## Abstract

This study investigated the comparative reinforcement effect of *Achatina fulica* snail shell nanoparticles, montmorillonite, and kaolinite nanoclay on greenpoxy. Greenpoxy nanocomposites of snail shell nanoparticles, montmorillonite, and kaolinite nanoclay were developed separately, with the nanofiller content ranging from 1 to 3% by weight. Specimens of the nanocomposites with different percentage weights of the nanoparticles were prepared using the resin casting method. Mechanical properties, such as the tensile strength, stiffness, hardness, and impact strength, and water absorption properties of the specimens were evaluated experimentally. It was observed that the incorporation of nanoparticles improved the mechanical properties of pure greenpoxy irrespective of the percentage weight, source, and type of reinforcement. Significantly, the loading of 1 wt.% of snail shell nanoparticles offered superior properties in most cases. Protein fibers and high-concentration calcium carbonate in snail shell nanoparticles, uniform dispersion, and excellent matrix/snail shell nanoparticle adhesion provided a strong structure, resulting in the high strength, stiffness, and decreased water uptake of the composites. The superior properties observed in snail shell nanoparticle composites suggest that this naturally sourced nanofiller can be used as a potential substitute for montmorillonite and kaolinite clays.

## 1. Introduction

Reinforcement fillers of different particle sizes have been extensively incorporated in order to improve polymeric materials’ strength and stiffness. Several composite materials with different combinations of fillers and polymers (thermoplastic and thermoset) have been developed in this process. These developed composite materials have been chosen for several applications in the industry [1,2]. The wide application of a bionanocomposite can result from its easy processability and improved properties [3,4]. Several studies have established the significant role of loading fillers in improving the properties of polymeric materials [1,5,6]. In particular, commercial fillers such as carbon-based fillers (calcium carbonate CaCO_3,_ carbon nanotube, graphite), talc, montmorillonite, and kaolinite have been widely used to improve polymeric properties, which helps to reduce the cost of the expensive polymetric matrix and eventually reduces production costs [7,8,9,10,11,12,13]. Among the filler materials mentioned above, carbon-based reinforcement materials such as calcium carbonate CaCO_3_ are the most used because they are readily available and exhibit excellent thermal and mechanical strength with good reinforcement properties [3,4]. This carbon-based material is obtained from different sources, including rock, human, and animal waste, using various nanotechnology methods, and their particle sizes and chemical and thermal properties have been evaluated. These fillers’ improved chemical, thermal, and mechanical properties have resulted in their wide use as fillers or reinforcements in composites, papers, and paints [14,15,16].

Talc, montmorillonite, and kaolinite nanoclays are produced from rock through either explosion or gas pressure blasting techniques [17,18,19]. The pulverized materials are then milled to produce nanoparticles. Nanoparticles can be produced using precipitation techniques by reacting calcium hydroxide with carbon dioxide or grinding using available grinding machines. Particles produced using precipitation are often smaller than particles produced using the grinding method, and their application enhances the mechanical properties of composite materials because they have a good surface area [20,21]. Despite the excellent reinforcement effectiveness of talc, montmorillonite, and kaolinite nanoclays, they contain some toxic elements that are harmful to human health. Moreover, mining of the materials is not desirable and is considered unsustainable. Hence, there is a need for sustainably resourced fillers. This disadvantage has led to an innovative means of producing CaCO_3_ filler from agricultural waste, especially snail shells, using different techniques [15,22]. Several studies have investigated the chemical, physical, and reinforcement of calcium carbonate of different particle sizes produced for naturally sourced materials [23,24,25,26]. However, these studies only focus on determining the reinforcement effects of calcium carbonate-based fillers on polymeric properties individually. Significantly, limited studies compare the reinforcement effects of naturally sourced and commercial calcium carbonate-based fillers on polymer properties.

Syamimi et al. [27] explored the heat treatment influence of snail shell particles on a snail-shell-filled epoxy composite’s mechanical and thermal properties. The epoxy was reinforced with snail shell particles ranging from 5–15 wt.%. The mechanical, thermal, structural, and morphological properties of the epoxy/snail shell particles were investigated. The results gathered proved that the loading of 10 wt.% heat-treated snail shell improved the mechanical properties. Furthermore, the loading of snail shells increased the glass transition and decomposition temperature of the epoxy. Based on these facts, this study proposed snail shell particles as a promising bio-filler for the development of biocomposites.

Moreover, several studies have investigated the effect of loading eggshell particles (micro/nanoparticles) on polymeric material properties [28,29,30,31]. The outcomes of these studies proved that the loading of eggshell particles might be a viable means of modifying the properties of polymeric materials. Onuegbu and Igwe [32] compared the effect of snail shell and talc concentration and particle sizes on the mechanical properties of polypropylene. The results proved that the mechanical properties decrease with an increase in filler content, and superior properties were observed after loading the smallest particle sizes of snail shells [32]. However, a microparticle of unknown species of snail shell was used, and the study was only limited to a thermoplastic polymer. Our previous studies determined the *Achatina Fulica* shell’s reinforcement effectiveness [33,34,35]. The comparative studies on the properties of achatina fulica snail (S-shell) and eggshell particle (E-shell) composites proved that the loading of CaCO_3_ produced from *Achatina Fulica* offered improved mechanical properties in the polymer compared to eggshell particles [33]. However, this study was limited to CaCO_3_ sourced from natural or agricultural waste only. Thus, the reinforcement effect of commercial and CaCO_3_ synthesized from *Achatina Fulica* shells needs to be compared. This comparative study is necessary to determine whether CaCO_3_ synthesized from *Achatina Fulica* shell could serve as alternative filler material to commercial CaCO_3_ for composite, biocomposite, and nanocomposite development. Hence, this present study investigated and compared the thermo-mechanical properties of bionanocomposites separately filled with nano-CaCO_3_ synthesized for *Achatina fulica*, montmorillonite, and kaolinite.

## 2. Experimental Details

### 2.1. Raw Materials

Greenpoxy and catalyst (SR 33 and SD 4775) with high carbon content from plant origin were used as the bio-based binder. The greenpoxy used in this study was a sustainably sourced, high-performance, biobased polymer derived from plant biomass. An in-house synthesizing nano-CaCO_3_ from *Achatina fulica* shell through mechanochemical techniques, montmorillonite, and kaolinite provided by CSIR South Africa was used for the nanoparticles. The particle size of the nanoparticles used for the study was ≤100 nm.

### 2.2. Procedure for Synthesizing Nano-CaCO_3_ from *Achatina fulica* Shell

*Achatina fulica* shells were collected from a snail farm and were soaked in 6% sodium hypochlorite solution for 6 h. These shells were removed from the sodium hypochlorite solution and dried at room temperature for 24 h before milling. Dried shells were dry-milled using a planetary ball mill (Retsch^®^ PM 100) (Hamburg, Germany) to obtain a fine particle [28,36]. This process was achieved by milling snail shells at 450 rpm for 30 min in a clockwise direction and then sieving them using a mechanical sieving shaker (Retsch, AS 200 basics, Hamburg, Germany) to a size of ≤50 µm. These powdered snail shells were further wet-milled to achieve nanoparticles. Then, 30 g of snail shells with a particle size of ≤50 µm was measured into the 500 mL; after this, 100 mL of ethanol was added and the mixture was wet-milled at 450 rpm for 258 min in a clockwise direction. Afterward, mixtures of fine particles and ethanol were produced using the decantation method. The collected particles were washed by adding distilled water and separated using the decantation method to remove the remaining solvent. This process was repeated five times to ensure the purity of the fine particles. Then, particles were oven-dried at 35 ℃ for 72 h.

### 2.3. Preparation of Nanocomposites

The nanocomposite was developed using the conventional resin casting technique. Nanoparticles were uniformly dispersed separately in the greenpoxy using a magnetic stirrer with a hotplate. The viscosity of greenpoxy was reduced by measuring 100 wt.% (70 g) greenpoxy resin into a beaker using a digital electronic scale of 0.001 g; then, it was placed on the hotplate and heated up to 50 °C. The heating process was monitored for 30 min using a temperature probe. This process was carried out to facilitate the uniform dispersion of the nanoclay. Nanoparticles from snail shell montmorillonite and kaolinite (1–3 wt.%) were added separately into the greenpoxy and mixed using a mechanical stirrer at 500 rpm for one hour to ensure homogeneous dispersion. The loading of nanoparticles was kept low because it has been proven that nanoparticles are significantly effective at low loading [13,37]. Subsequently, the greenpoxy and nanoparticle blend was removed from the stirrer and cooled down to ambient temperature. The catalyst was then added to the nanoparticle/greenpoxy blend at a mixing ratio of 100:27 wt.% to facilitate the curing process.

A releasing agent (wax) was applied on the inner surface of the open plastic mold to facilitate easy removal of the composite panel. The nanocomposite blend was then poured into the mold to develop a composite panel and allowed to cure for two days. The cured nanocomposite was removed from the mold after 48 h and adequately cured for another 15 days. Then, the physical and mechanical properties of the developed nanocomposite panel were evaluated afterward.

### 2.4. Testing of Composite Characteristics

#### 2.4.1. Tensile Strength

The tensile properties were evaluated to determine the pulling strength and stress resistance of the nanocomposites (strength and stiffness). The evaluation was carried out on a Lloyd universal testing machine (Model 43) fitted with a 30 kN load cell manufactured by MTS (Eden Prairie, Minnesota, MN, USA). The test was carried out using the ASTM 3039 test standard. Test samples of 250 mm × 25 mm × 3 mm dimensions were cut from the neat greenpoxy and the nanocomposite laminate using a computer numerical control (CNC) machine to ensure the exact dimensions of the testing samples. Five samples were tested at room temperature, using a constant testing cross-head speed of 1 mm/min. The mean value of the five samples was used for graphic illustration and discussion.

#### 2.4.2. Hardness Test

The hardness property was investigated to determine the nanocomposite’s resistance to indentation. This test was conducted according to the ASTM D 2583 test standard using a Barcol hardness tester (TX Testing instrument, Shenyang, China). A Barcol hardness tester is a piece of standard equipment that is impressed with a steel truncated cone (6.82 mm) and a tip diameter of 0.55 mm, and it was used at a 26° angle. This indenter was placed on the upper flat surface of the nanocomposite panel, a uniform descending press was applied by hand, and readings were collected directly from the dial gauge. Twenty-five indentation readings were randomly collected on each specimen, and the mean values were reported.

#### 2.4.3. Water Absorption

Water absorption of the nanocomposites was determined by immersing rectangular samples 10 mm × 5 mm × 3 mm in water for 24 h at room temperature. This test was conducted according to ASTM D570-98 standard test specifications. The initial weight of five nanocomposites with different nanofillers was taken before immersion in water. Then, samples were dipped into water at room temperature. Subsequently, the sample was removed, wiped with a dry napkin, and weighed to determine the sample’s final weight (W2) using a Sartorius digital electronic scale with 0.001 g accuracy (Model BP-1108) made in Göttingen, Germany. The following equation was used to determine the rate of absorption of the nanocomposite as a percentage.

(1) $$f_{B}=\frac{f_{2}-f_{1}}{f_{1}}\times 100$$

where $f_{B}$ is the percentage of absorption. Five samples were tested, and the average $f_{B}$ value of the five samples was illustrated and considered.

#### 2.4.4. Impact Resistance

The impact resistance of the nanocomposites was determined according to ASTM D6110-10 at room temperature using the Charpy test performed with an Unnotched Izod impact machine (Tensiometer Ltd., Croydon, UK). Five biocomposite test specimens were tested, and the mean value is considered in the Results and Discussion.

#### 2.4.5. Scanning Electron Microscopy

The fracture mechanism that governed the nanocomposite’s mechanical properties was determined by scanning electron microscopy. The fracture surface of the nanocomposite was sputter-coated at 25 mA using a Quorum K550x gold sputter coater and then observed on a Phenom Pharos desktop SEM (Thermo Fisher Scientific, Albany, Auckland, New Zealand). The SEM images were captured at an accelerating voltage of 10 kV.

## 3. Results and Discussion

### 3.1. Tensile Properties

**Figure 1:**
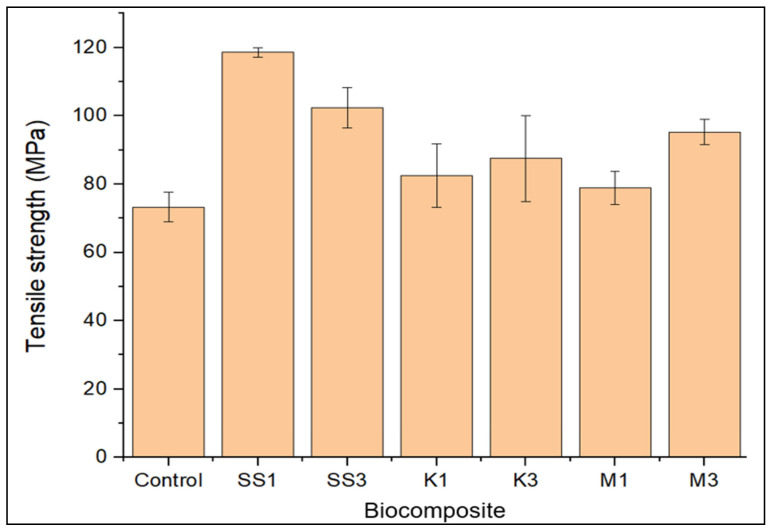
Tensile strength of unfilled (control), snail shell nanoparticle—(SS), montmorillonite—(M), and kaolinite—(K) filled greenpoxy nanocomposite.

**Figure 2:**
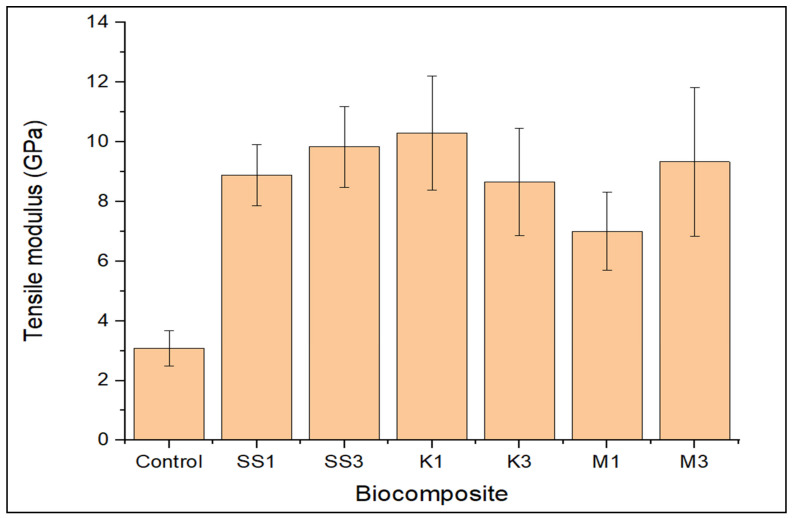
Tensile stiffness of unfilled (control), snail shell nanoparticle- (SS), montmorillonite- (M), and kaolinite- (K) filled greenpoxy nanocomposite.

Nanocomposite strength and stiffness data, shown in Figure 1 and Figure 2, compare the effect of loading snail shell nanoparticles, montmorillonite, and kaolinite clay samples on the tensile properties of unfilled greenpoxy. For tensile strength, as shown in Figure 1, the loading of nanofillers increased the strength of unfilled greenpoxy irrespective of the type and source. This output may be attributed to the reinforcement effect of the nanoparticle incorporated. It also confirmed the positive reinforcement effects of the snail shell nano-CaCO_3_, montmorillonite, and kaolinite as reported in the available literature [26,37,38,39,40,41].

Although the loading of the nanofillers enhanced the tensile strength of greenpoxy, the nanocomposite with snail shell nanoparticles exhibited greater tensile strength than the montmorillonite- (M) and kaolinite- (K) filled epoxy nanocomposites. Furthermore, the error bars presented in all the figures reported are the standard deviation. This significant increase in tensile strength may result from the large volume of carbon in the snail shell nanoparticles compared to montmorillonite, as shown in Table 1, which improves the adhesion at the matrix and the filler interfacial surfaces, resulting in higher tensile strength. The reinforcement potential of montmorillonite was greater than that of kaolinite. This observation may be attributed to the reinforcement effectiveness of carbon-based fillers (snail shell nanoparticles and montmorillonite), known for transferring atoms, forming an interlocking structure that results in improved tensile strength in the nanocomposite [13,39,41]. This behavior also confirmed the available literature in which the loading of carbon-based fillers increased the polymer strength better than SiO_2_-based fillers [42]. In this study, the nanocomposite with 1 wt.% snail shell nanoparticles is denoted as SS1, while SS3 symbolizes the nanocomposite with 3 wt.% snail shell nanoparticles. M1 represents the nanocomposite with 1 wt.% montmorillonite, and the biocomposite with 3 wt.% montmorillonite is denoted as M3. Similarly, K1 represents the nanocomposite with 1 wt.% kaolinite and K3 symbolizes the nanocomposite with 3 wt.% kaolinite.

Furthermore, the chemical composition, source, and different loading of these fillers may explain the random effects of their loadings in the composites on the strength and stiffness of the biobased polymer. The loading amount of nanofiller improved the greenpoxy strength and stiffness differently. It was observed that the addition of 1 wt.% snail shell nanoparticles (*SS1*) enhanced the strength of greenpoxy by 62%, which was higher than the 8 % and 13 % tensile strength improvement observed after loading the same percentage of montmorillonite and kaolinite, respectively. This output proves the effectiveness of snail shell nanoparticles on the strength of greenpoxy at low loadings [13,37]. The homogeneous dispersion of nanoparticles in the matrix, forming an interlocking structure, may also account for the improved tensile strength observed at a low loading of the snail shell nanoparticles [14]. This trend agrees with literature results in which low loadings of the nanoparticle improved the mechanical properties of the polymeric material [8,43,44]. It is well known that natural resources such as snail shells possess protein fibers that make them very rigid, resulting in resistance to stress and indentation, implying that the nanoparticles’ source also has a significant effect on their reinforcement properties. The protein fibers in snail shell nanoparticles may also be the reason for the improved tensile strength observed by increasing the snail shell nanoparticle composite’s resistance to pulling stresses [35,45,46].

Concomitantly, the filler and matrix compatibility may be a reason for the enhanced strength of the nanocomposites. Both the polymer and snail shell nanoparticles were sourced from the same carbon-based material, making them compatible. This compatibility results in excellent adhesion at the interface of the nano-CaCO_3_ and greenpoxy, producing more consistent structures with excellent resistance to external pulling stress.

Considering the loading of nanoparticles at 3 wt.%, nanocomposite *SS3* with snail shell nanoparticles exhibited higher tensile strength. The nanocomposite strength enhancement can be traced to the high surface area of the carbon-based nanoparticles and their adhesion to the matrix. The alkaline treatment undergone by the snail shell before milling may be another reason for the enrichment in strength observed. It is well known that chemical treatment often removes impurities, enhancing the adhesion capability of the filler material. This filler material adhesion eventually provides strong adhesion between the nanoparticle and polymeric molecules at the interface, leading to a structural formation with good resistance to pulling stresses.

Furthermore, for the stiffness result shown in Figure 2, it was observed that the loading of all the nanoparticles incorporated improved greenpoxy stiffness. The positive influence on greenpoxy stiffness proved that the loading of snail shell nanoparticles, montmorillonite, and kaolinite improved the strength and effectively improved the stiffness of the greenpoxy. This trend is consistent with literature reports where the loading of carbon- and SiO_2_-based fillers improved the strength and stiffness of the composite material [8,22,42]. It was further observed that the stiffness properties of greenpoxy increased with a corresponding increase in snail shell nanoparticles, and the same trend was observed with the loading of montmorillonite. This performance may be attributed to covalent bonding and *catenated* carbon structures that can transfer atoms in the matrix as greenpoxy and nanoparticles are carbon-based materials. The combination of these two materials formed an interlocking structure with improved strength—the nanocomposite with 1 wt.% kaolinite (K1) exhibited higher tensile stiffness (10.2975 GPa) than composites with snail shell nanoparticles and montmorillonite, which may be attributed to homogeneous dispersion. However, an insignificant difference in stiffness was exhibited in composites with snail shell nanoparticles and montmorillonite. This performance may result from the inherent stiffness properties of the nanoparticles incorporated.

**Figure 3:**
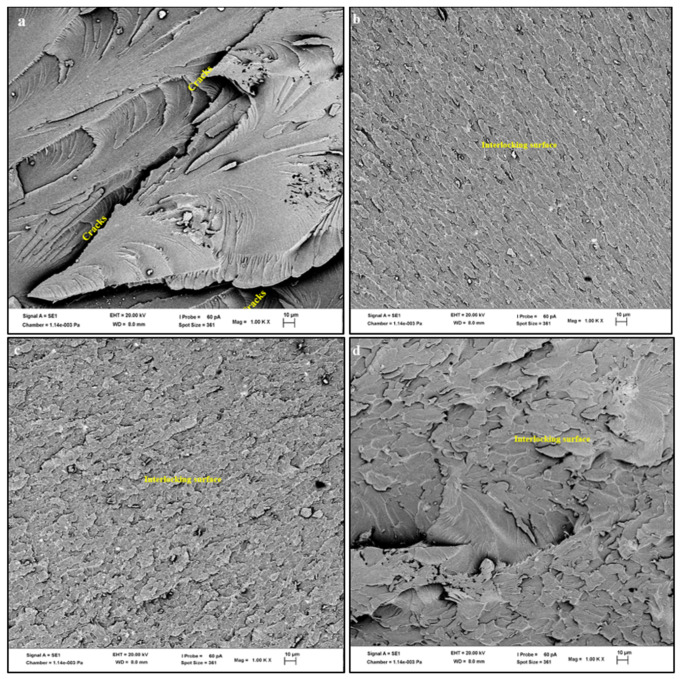

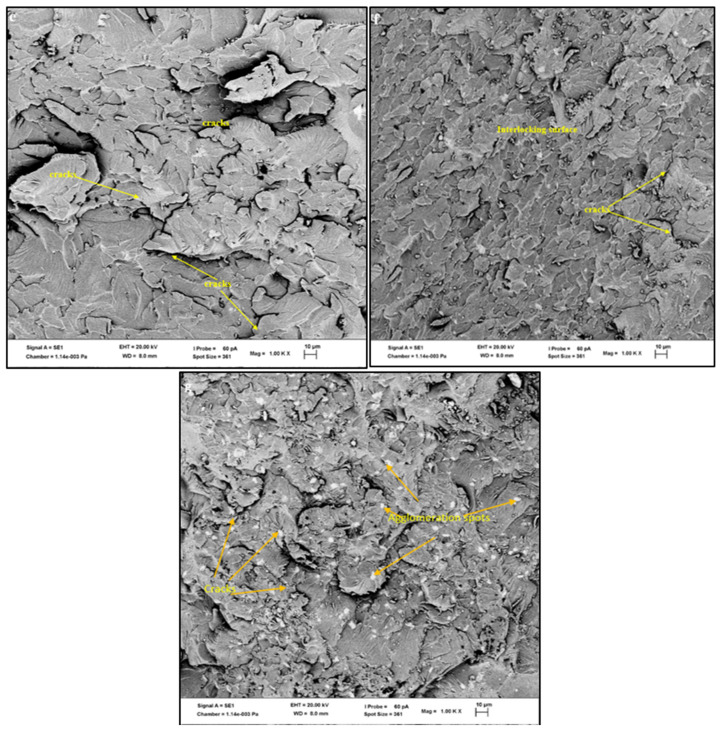
SEM micrographs showing tensile fractography of (**a**) neat greenpoxy, (**b**) SS1-, (**c**) SS3-, (**d**) K1-, (**e**) K3-, (**f**) M1-, (**g**) M3-filled greenpoxy nanocomposite.

The SEM fracture surface images for greenpoxy and the snail shell nanoparticle-, montmorillonite-, and kaolinite-filled greenpoxy nanocomposite are shown in Figure 3. A relatively smooth plateau with cracks, indicating a brittle fracture, can be seen on the neat greenpoxy fracture surface in Figure 3a. This fracture mechanism may be attributed to the brittle properties associated with all polymeric materials. An interlocking texture structure, cracks, and particle agglomeration are evident on the snail shell nanoparticle-, montmorillonite-, and kaolinite-filled greenpoxy nanocomposite fracture surfaces. It is known that the incorporation of fillers often influences the structure of the polymer, which may improve or inversely affect the final material properties [4,43].

Accordingly, the incorporation of 1 wt.% and 3 wt.% nanoparticles had different effects on the greenpoxy structure. It was observed that 1 wt.% and 3 wt.% loadings reduced crack propagation in pure greenpoxy, which improved its mechanical and water-permeable barrier properties. Nanocomposites with 1 wt.% and 3 wt.% snail shell nanoparticles (Figure 3b,c) revealed fracture surfaces with a homogeneous nanofiller distribution without any cracks. The compatibility and good adhesion of matrix–shell particles at the interface provided a structure that helped to increase the strength and may be related to the improved mechanical properties observed in Figure 1, Figure 2, Figure 5, and Figure 6.

**Figure 4:**
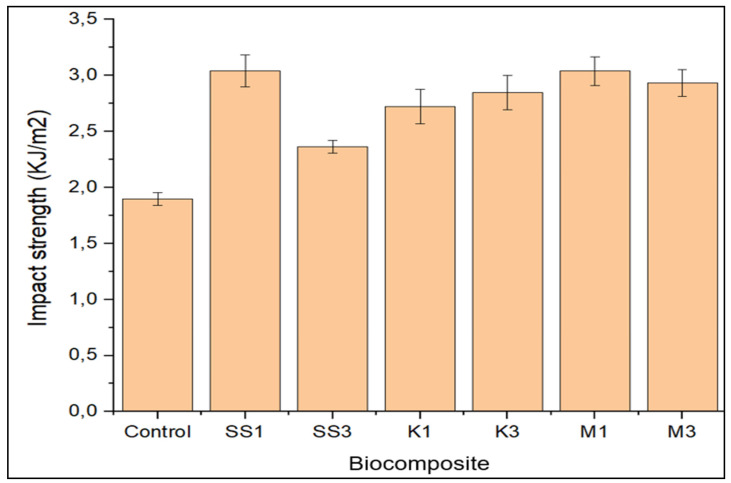
Impact strength of unfilled (control), snail shell nanoparticle—(SS), montmorillonite—(M), and kaolinite—(K) filled greenpoxy nanocomposite.

Furthermore, the inclusion of snail shell nanoparticles probably induced crack arresting and a pinning mechanism in the matrix at the initial stage, serving to block crack propagation [13,33,35]. A more rigid surface texture and tiny cracks were seen on the fracture surface of the 1 wt.% kaolinite-reinforced nanocomposite shown in Figure 3d. This SEM image also shows the uniform dispersion of nanoparticles as it is challenging to see nanoparticles on the fracture surface. This strict structure may have provided internal stiffness, which eventually reduced plastic deformation and resisted shock and water permeation, resulting in improved stiffness, impact resistance, and low water uptake, as seen in Figure 2 and Figure 4.

Similarly, the fracture surface of the nanocomposite with 1 wt.% montmorillonite revealed an interlocking structure with a homogeneous dispersion of nanoparticles and limited tiny cracks, which supported the improved mechanical properties observed. The fracture surface of the nanocomposite with 3 wt.% was dominated by microcracks and agglomeration spots; meanwhile, for the nanocomposite reinforced with shell snail nanoparticles, no cracks or agglomeration were seen.

The agglomeration of particles observed on the fracture surface of M3 and K3 in Figure 3e,f resulted from incorporating a larger concentration of nanoparticles, leading to microcracks. High loading of nanoparticles in a matrix often reduces the required amount of polymer at the nanoparticle interface, which reduces interfacial bonding, producing a weaker structure that may not resist external stresses. Consequently, higher amounts of particles in the matrix induce stress concentrations, weakening the adhesion between particles and the matrix, resulting in cracking propagation and later failure. The weakened structure may be attributed to the reduced strength, stiffness, and water uptake observed for these nanocomposite series shown in Figure 2 and Figure 3.

### 3.2. Impact Strength

Results for the impact strength of unfilled and nanoparticle-filled greenpoxy are represented in Figure 4. It was observed that the impact resistance of greenpoxy was improved with nanoparticle loading, irrespective of the type and loading percentage; however, the nanocomposite with 1 wt.% snail shell nanoparticles offered superior impact resistance. The significant improvement in strength may be attributed to the interconnecting bond formed by the filler and polymeric material, which eventually improved the energy-absorbing ability. The nanoparticle-reinforced greenpoxy nanocomposites with 1 wt.% snail shell nanoparticles and montmorillonite exhibited nearly the same impact resistance of 3.041 KJ/m^2^ and 3.039 KJ/m^2^. This improvement is around 60% greater than 1.89918 KJ/m^2^ observed for pure greenpoxy. This output demonstrates the reinforcement influence of snail shell nanoparticles and montmorillonite at low concentrations on the impact resistance properties of greenpoxy. Although 1 wt.% loading of kaoline improved neat greenpoxy by 43.3%, the nanocomposite with SS and M offered higher impact resistance than the nanocomposite with K.

This performance can be attributed to the inherent covalent bonding of the carbon-based fillers, which enhances the inner adhesion of the nanoparticle and greenpoxy molecules, resulting in a material structure with good impact resistance [33,34,35]. A drop in impact strength was observed when the loading of SS and M increased to 3 wt.%. This decrease in impact resistance may be ascribed to the agglomeration formed after incorporating a high volume of nanoparticles. After loading snail shell nanoparticles (SS), the improvement in the tensile and impact properties of greenpoxy proved that this natural-based filler material has reinforcement efficiency and could serve as an alternative to commercial filler montmorillonite (M).

### 3.3. Hardness Property

**Figure 5:**
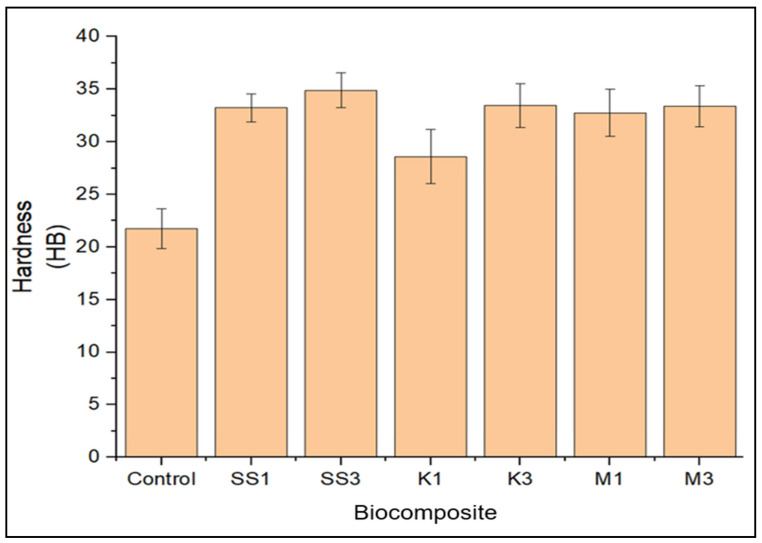
Hardness property of unfilled (control), snail shell nanoparticle—(SS), montmorillonite—(M), and kaolinite—(K) filled greenpoxy nanocomposite.

The hardness property of the unfilled, snail shell nanoparticle-, montmorillonite-, and kaolinite-filled greenpoxy nanocomposites is illustrated in Figure 5. The developed nanocomposites were subjected to hardness property evaluation to determine their resistance to indentation. It was observed that the hardness property of greenpoxy increased after the addition of nanoparticles, irrespective of the loading weight percentage. This hardness improvement may be attributed to the admirable dispersion and superior adhesion of the nanoparticles and matrix. At 1 wt.% loading, snail shell nanoparticles increased greenpoxy’s hardness by 53%, which is greater than the hardness value observed after incorporating montmorillonite and kaolinite. However, the nanocomposite with 1 wt.% montmorillonite exhibited higher hardness than the 1 wt.% kaolinite-reinforced greenpoxy nanocomposite. This trend corresponds with the impact properties observed in Figure 4.

This notable hardness improvement may be attributed to the interlocking structure formed through an adhesion bond that was dominant between the filler and matrix, resulting in a stringent surface that resisted indentation—with an insignificant linear increase in hardness properties as the nanoparticle loading increased to 3 wt.%, except for the composite with kaolinite, with a marginal difference. However, the comp.osite with 3 wt.% snail shell nanoparticles exhibited higher hardness properties. This trend is consistent with studies in which increasing the loading of nanoparticles increased the hardness property of the polymer [13,33,45].

### 3.4. Water Uptake

**Figure 6:**
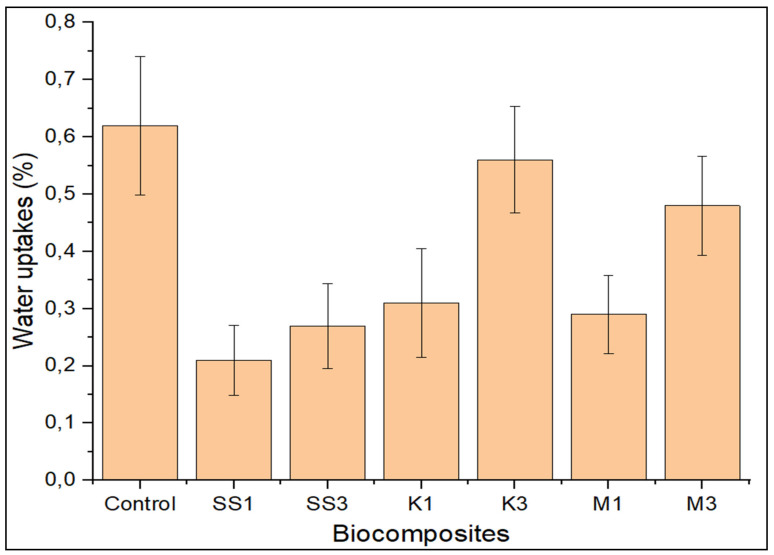
Water uptake of unfilled (control), snail shell nanoparticle—(SS), montmorillonite—(M), and kaolinite—(K) filled greenpoxy nanocomposite.

The water uptake (WU) for the pure greenpoxy, snail shell nanoparticle-, montmorillonite-, and kaolinite-filled greenpoxy nanocomposites is shown in Figure 6. It was observed that the incorporation of snail shell nanoparticles, montmorillonite, and kaolinite nanoparticles at low concentrations of 1 wt.% significantly decreased the absorption of greenpoxy. The hydrophobic nature of the nanoparticles served as a barrier for water permeation into the nanocomposite. Homogeneous dispersion in a small concentration of nanoparticles may be another reason for the reduced absorption rate observed. Furthermore, a sharp drop in the water absorption rate after introducing nanoparticles may have been attributed to closer packing from the homogenous dispersion of the nanoparticles, which produced a more rigid structure that resisted water penetration. This trend is consistent with the literature in which the loading of nanoparticles reduced water uptake [13,33].

A significant increase was observed as the loading of snail shell nanoparticles, montmorillonite, and kaolinite increased to 3 wt.%. This increase in WU may be attributed to the relative increase in the loading of nano-CaCO_3_, forming a particle agglomeration structure with weak resistance to water uptake. However, the nanocomposite with 1 wt.% snail shell nanoparticles exhibited the lowest absorption rate. This performance could be attributed to the higher concentration of carbon present in the snail shell, as shown in Table 1. The hydrophobic nature of the carbon-based filler and the interrelating bonds formed at the interface of the filler and matrix resulted in a WU reduction. The chemical treatment given to the fibers and nano-CaCO_3_ may be another reason for the reduction in water uptake.

## 4. Conclusions

The reinforcement effect of *Achatina fulica* snail shell nanoparticles, montmorillonite, and kaolinite nanoclays on the mechanical properties and water uptake were successfully investigated. The loading of snail shell nanoparticles, montmorillonite, and kaolinite nanoclay improved pure greenpoxy’s properties irrespective of the concentration and source. Incorporating 1 wt.% nanoparticles led to higher tensile strength and stiffness, higher impact, and lower water uptake properties compared to 3 wt.% loading. This performance proves the effectiveness of adding low nanofiller concentrations on improving greenpoxy’s properties. Superior properties were observed in most cases after the addition of 1 wt.% snail shell nanoparticles. This performance was attributed to the inherent properties, source, and uniform dispersion of the incorporated nanoparticles, leading to outstanding matrix/nanofiller adhesion, resulting in a stronger nanocomposite with improved properties. An insignificant linear improvement in the hardness property was noted with a corresponding increase in nanoparticle loading from 1 wt.% to 3 wt.%. However, the nanocomposite with 1 wt.% snail nanoparticles displayed noticeably higher tensile strength and stiffness, impact strength, water barrier, and significantly high hardness properties. Adverse effects such as agglomeration and weak adhesion at the matrix and particle interface were observed at 3 wt.% loadings of montmorillonite, and kaolinite nanoclay resulted in a drop in the properties of these nanocomposite series. The overall findings suggest that snail shell nanoparticles possess reinforcement potential and could serve as an alternative montmorillonite and kaolinite nanoclay.

## Figures and Tables

**Table 1:** Elemental composition of snail shell nanoparticles (SS3), montmorillonite (M3), and kaolinite (K3).

**Snail shell nanoparticles (SS)**	***Elemental composition***	**C**	**O**	**Ca**	**_**	**_**	**_**
	***Wt.%***	**36.71**	**22.25**	**40.44**	**_**	**_**	**_**
**Montmorillonite (M)**	***Elemental composition***	**O**	**C**	**Ca**	**Al**	**Te**	**Nb**
	***Wt.%***	**46.79**	**25.18**	**11.48**	**12.38**	**2.74**	**3.18**
**Kaolinite (K)**	***Elemental composition***	**O**	**Si**	**Al**	**Mg**	**Sr**	**Na**
	***Wt.%***	**48.05**	**30.43**	**9.23**	**2.76**	**7.69**	**1.76**

## Data Availability

Not applicable.
